# Precision therapy for ulcerative colitis: insights from mitochondrial dysfunction interacting with the immune microenvironment

**DOI:** 10.3389/fimmu.2024.1396221

**Published:** 2024-07-04

**Authors:** Yi-fan Zhang, Meng-ying Fan, Qi-rui Bai, Rong Zhao, Shan Song, Li Wu, Jun-hui Lu, Jing-wei Liu, Qi Wang, Yuan Li, Xing Chen

**Affiliations:** ^1^ The First Clinical Medical College, Shanxi Medical University, Taiyuan, China; ^2^ Department of Gastroenterology, The First Hospital of Shanxi Medical University, Taiyuan, China; ^3^ The Anesthesiology College, Shanxi Medical University, Taiyuan, China; ^4^ The Second Clinical Medical College, Shanxi Medical University, Taiyuan, China; ^5^ School of Basic Medical Sciences, Shanxi Medical University, Taiyuan, China

**Keywords:** ulcerative colitis, mitochondria, immune infiltration, metabolism, bioinformatics analysis, machine learning, unsupervised clustering

## Abstract

**Background:**

Accumulating evidence reveals mitochondrial dysfunction exacerbates intestinal barrier dysfunction and inflammation. Despite the growing knowledge of mitochondrial dysfunction and ulcerative colitis (UC), the mechanism of mitochondrial dysfunction in UC remains to be fully explored.

**Methods:**

We integrated 1137 UC colon mucosal samples from 12 multicenter cohorts worldwide to create a normalized compendium. Differentially expressed mitochondria-related genes (DE-MiRGs) in individuals with UC were identified using the “Limma” R package. Unsupervised consensus clustering was utilized to determine the intrinsic subtypes of UC driven by DE-MiRGs. Weighted gene co-expression network analysis was employed to investigate module genes related to UC. Four machine learning algorithms were utilized for screening DE-MiRGs in UC and construct MiRGs diagnostic models. The models were developed utilizing the over-sampled training cohort, followed by validation in both the internal test cohort and the external validation cohort. Immune cell infiltration was assessed using the Xcell and CIBERSORT algorithms, while potential biological mechanisms were explored through GSVA and GSEA algorithms. Hub genes were selected using the PPI network.

**Results:**

The study identified 108 DE-MiRGs in the colonic mucosa of patients with UC compared to healthy controls, showing significant enrichment in pathways associated with mitochondrial metabolism and inflammation. The MiRGs diagnostic models for UC were constructed based on 17 signature genes identified through various machine learning algorithms, demonstrated excellent predictive capabilities. Utilizing the identified DE-MiRGs from the normalized compendium, 941 patients with UC were stratified into three subtypes characterized by distinct cellular and molecular profiles. Specifically, the metabolic subtype demonstrated enrichment in epithelial cells, the immune-inflamed subtype displayed high enrichment in antigen-presenting cells and pathways related to pro-inflammatory activation, and the transitional subtype exhibited moderate activation across all signaling pathways. Importantly, the immune-inflamed subtype exhibited a stronger correlation with superior response to four biologics: infliximab, ustekinumab, vedolizumab, and golimumab compared to the metabolic subtype.

**Conclusion:**

This analysis unveils the interplay between mitochondrial dysfunction and the immune microenvironment in UC, thereby offering novel perspectives on the potential pathogenesis of UC and precision treatment of UC patients, and identifying new therapeutic targets.

## 1 Introduction

Ulcerative colitis (UC) is an idiopathic chronic inflammatory bowel disease (IBD) characterized by mucosal inflammation, which starts in the rectum and generally extends continuously to proximal segments of the entire colon (1). The global incidence and prevalence of UC have been increasing, affecting millions of patients across the globe (2). Despite the exact etiology is not fully understood, it is regarded to be an interaction of multiple factors, involving genetic predisposition, epithelial barrier defects, microbiota, leucocyte recruitment, and dysregulated immune responses (3, 4). The diagnosis of UC is confirmed by clinical symptoms, endoscopic biopsy, and histological evaluation, and there is currently no gold standard (4). UC has been classified as proctitis, left-sided colitis or extensive pancolitis depending on the Montreal classification (5). Levels of disease severity - mild, moderate, or severe - is usually determined by the Mayo score (6) or Lichtiger score (7). Nevertheless, neither of above two clinical classifications, whether based on the inflammation extent or severity, takes into account the molecular mechanism of UC, which is a vital step forward in the drive towards precision medicine. Currently, the mainstay therapeutic agents for UC include 5-aminosalicylates, corticosteroids, immunomodulators and biologics, among which, biological agents, such as the tumor necrosis factor (TNF)-α inhibitors, infliximab (IFX) and Golimumab (GLM) (8, 9), anti-α4β7 integrin antibodies, vedolizumab (VDZ) (10) are the most classical and widely used medications for patients with UC, all of which can induce and maintain remission to promote mucosal healing (11). Additionally, another new drug, ustekinumab (an IL-12/IL-23 inhibitor) (12) has demonstrated efficacy in achieving clinical remission following the failure of anti-TNF-α therapy (11). Despite expanding therapeutic options, 10–20% of refractory patients still require proctocolectomy due to adverse drug reactions and secondary loss of response (4). The key to breaking through this therapeutic ceiling might be the combination of therapeutics with personalized precision therapy based on the identification of molecular subtypes that are unique to individual patients. Therefore, there is a pressing need to detect reliable diagnostic biomarkers and develop novel molecular stratification to approach more effective therapeutic strategies of UC patients.

Emerging research has recently revealed that mitochondrial dysfunction is implicated as a key factor in UC pathogenesis (13). Mitochondrial dysfunction can lead to energy deficiency to cause abnormal energy metabolism, such as tricarboxylic acid (TCA) cycle, fatty acid catabolism, and amino acid biosynthesis, often impair the epithelial barrier function by increasing susceptibility to TNF-α-induced cell death, reducing secretory barrier function (especially Paneth cells and goblet cells), and responding to regenerative capacity for damaging stimuli (14–16). Thus, the colonic epithelia of UC patients might have a uniquely high susceptibility to mitochondrial dysfunction, which can be revealed by the immune microenvironment of the colonic mucosa to affect the response to immunotherapy. Mitochondrial damage is able to affect the phenotype and activation of infiltrating immune cells, especially dendritic cells (DCs), macrophages, and B cells, via metabolic reprogramming, which contributes to the maintenance of an inflammatory milieu for abnormal innate and adaptive immune responses, then in turn further exacerbates mitochondrial dysfunction in the colonic mucosa (17, 18). A comprehensive comprehension of the mechanisms underlying mitochondrial action in UC may provide new insights into unraveling the complexity and heterogeneity of UC, facilitate the identification of optimal treatment strategies for UC patients for better outcomes, and aid in the discovery of novel therapeutic targets.

In our study, the most comprehensive colonic mucosal tissue transcriptomic data by integrating the publicly available UC transcriptome datasets to date were utilized to explore the mechanisms underlying mitochondrial action in UC through comprehensive bioinformatics analysis. Based on a variety of machine learning algorithms, UC genes diagnostic model was developed. Unsupervised clustering was applied to identify genes subclassification in UC patients to comprehensively characterize the molecular and clinical characteristics of the subtypes. Furthermore, the patients in different UC subclassification showed different performance in the efficacy of the four biological agents, infliximab (IFX), ustekinumab (UST), vedolizumab (VDZ) and golimumab (GLM), respectively. These may provide new ideas for the clinical precision therapy of UC patients from the perspective of disease heterogeneity.

## 2 Materials and methods

### 2.1 Data acquisition and processing

**Figure 1:**
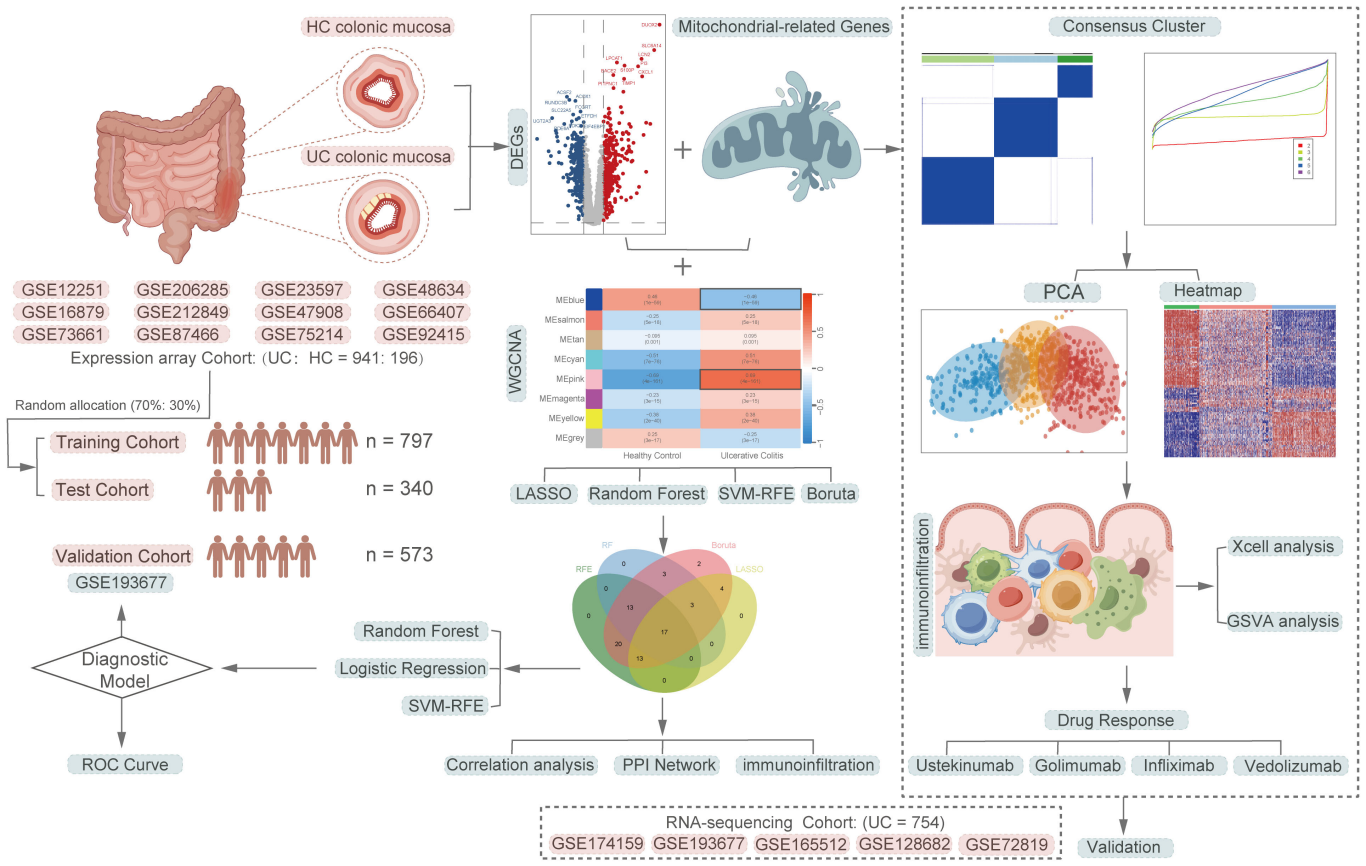
A comprehensive flowchart of the research procedure. Part of the cartoon graphical were drawn by Figdraw.

**Figure 1** depicts a comprehensive flowchart of the research procedure. The microarray and RNA-seq datasets analyzed in this study were obtained from the Gene Expression Omnibus (GEO, https://www.ncbi.nlm.nih.gov/geo/) database. The final participants included 17 independent UC datasets, comprising a total of 1891 samples, with 1695 UC samples and 196 healthy control samples. The training cohort consisted of 941 UC patients from 12 microarray datasets (GSE66407, GSE87466, GSE75214, GSE47908, GSE48634, GSE212849, GSE92415, GSE206285, GSE73661, GSE16879, GSE12251, GSE23597), while the validation cohort included 754 UC patients from 5 RNA-seq datasets (GSE174159, GSE193677, GSE165512, GSE128682, GSE72819). Detailed explanations for all datasets are presented in **Supplementary Table S1**. The mitochondria-related genes (MiRGs) were collected from MitoCarta 3.0 database (https://www.broadinstitute.org/mitocarta/mitocarta30-inventory-mammalian-mitochondrial-proteins-and-pathways) (19) and the gene set enrichment analyses (GSEA, http://www.gsea-msigdb.org/gsea/index.jsp)(**Supplementary Table S2**) (20, 21). For genes represented by multiple probes, only the probes with maximal signal were used for the further analysis. All microarray data were normalized using the “limma” R package (22). The raw count data from RNA-seq experiments were first transformed into the transcript per million (TPM) formats, followed by log2 (TPM+1) transformation, to achieve normalization. To remove batch effects of different data sources, the “ComBat” function within the “sva” R package (23) was utilized for batch correction.

### 2.2 Identification of differentially expressed mitochondria-related genes in UC

The “limma” R package was applied to identify differentially expressed genes (DEGs) between UC and HC samples (22). All analyses were adjusted for false positive results using False Discovery Rate (FDR) correction. Adjusted P-value < 0.05 and | logFC | > 0.58 were set as the threshold. Volcano plots and heat maps generated by the “ggpubr” and “pheatmap” packages, respectively, were used to visualize the screened DEGs. Additionally, Venn diagram was exploited to display common genes in both DEGs groups and MiRGs (24).

### 2.3 WGCNA and machine learning algorithms identified the diagnostic feature genes

Weighted correlation network analysis (WGCNA) is commonly used to reveal disease-related gene networks and identify co-expressed gene modules. To screen for co-expressed gene modules associated with UC, we performed WGCNA analysis using the “WGCNA” R package (25) for the top 25% of differentially ranked expressed genes. A soft threshold of β = 10 was selected for scale independence. The cluster height of module feature genes was set at 0.25, indicating a similarity greater than 0.75. Module and phenotypic data were utilized to evaluate gene significance (GS) and module significance (MS), and to investigate the correlation between modules and models. The most significant module genes, positively and negatively correlated with UC, were chosen for further analysis. To achieve the most effective feature subset, four machine learning algorithms were employed for feature selection: the Boruta algorithm, least absolute shrinkage and selection operator (LASSO), recursive feature elimination (RFE), and random forest (RF). The “Boruta” R package was used for the Boruta feature selection analysis, with 1000 significant runs (maxRuns = 1000). The LASSO algorithm, implemented through the “glmnet” R package (26), utilizes λ (lambda) as a penalty value or shrinkage operator to generate a penalty function. This function compresses the regression coefficients of variables in the model, effectively addressing the covariance issue and mitigating overfitting. The optimal composition of signature genes is determined by selecting the level of minimum cross-validation prediction error (lambdam.min). The backward feature elimination technique, known as RFE, is employed to eliminate feature vectors generated by SVM in order to identify the most optimal variables (27). In this study, we utilized the “caret” R package to implement the RFE algorithm for feature gene ranking and dimensionality reduction. Additionally, the “randomForest” R package (https://cran.r-project.org/web/packages/randomForest/) was utilized to construct the random forest tree of the feature genes, calculate their importance scores, and rank them accordingly. Only genes identified by the aforementioned four machine learning feature selection algorithms were deemed as key genes for subsequent analysis.

### 2.4 Construction and validation of a UC diagnostic model based on key MiRGs

A total of 1137 samples (UC: HC = 941:196) were randomly divided into a training set (UC: HC = 659:138) and a test set (UC: HC = 282:58) using the “caret” R package at a 70% to 30% ratio. The training cohort was balanced using the SMOTE algorithm from the “smotefamily” R package to address data imbalance and prevent overfitting. SMOTE is an oversampling method that creates new minority instances based on existing minority instances using the k-nearest neighbor algorithm. Logistic regression (LR), RF, and support vector machine (SVM) were employed to develop a diagnostic model in the training cohort. The LR algorithm was implemented using the “glm” function from the R package “glmnet”. To mitigate the risk of overfitting, a 10-fold cross-validation approach was employed. Furthermore, the model’s robustness was assessed by validating it in both the test cohort and an independent RNA-seq dataset, serving as an external validation cohort.

### 2.5 Molecular subtypes of UC driven by genes associated with mitochondria

To enhance our comprehension of the molecular attributes of genes associated with mitochondria in UC, we attempted to uncover the molecular heterogeneity associated with mitochondria in UC. Unsupervised consensus clustering algorithm analysis of 1000 iterations based on the identified MiRGs in the training cohort via R package “ConsensusClusterPlus” (28). The clustering algorithm was configured to employ the “km” method, while the similarity between samples was assessed based on the euclidean distance. The determination of the optimal number of clusters was based on the cumulative distribution function (CDF) and the relative change in the area under the CDF curve. Principal component analysis (PCA) plots were generated using the “ggord” R package to verify the consensus clustering outcomes. The expression of MiRGs in different subtypes was visualized using the R package “pheatmap”.

### 2.6 Cibersort and Xcell immune cell infiltration analysis

The immune infiltrating cells within the immune microenvironment of the colonic mucosa of patients with UC and HC were analyzed using the Cibersort algorithm (29). The infiltrating abundance of 22 immune cells infiltrating were evaluated and compared through the utilization of the Wilcoxon test. Additionally, the Spearman correlation was employed to investigate the associations between the expression levels of the identified core genes and the Cibersort scores of the 22 immune infiltrating cell types. To investigate the infiltration of cells in the immune microenvironment of the colonic mucosa in patients with UC, we employed Xcell analysis (30). This analysis utilized xCell’s standard 64 cell type signatures and was conducted through the “xCell” R package. The objective was to ascertain the abundance score of infiltrating immune cells and stromal cells in colonic mucosal tissues of distinct ulcerative colitis subtypes driven by mitochondrial genes.

### 2.7 Enrichment analyses and gene set variation analysis

In order to ascertain potentially enriched pathways for DEGs linked to mitochondrial disorders in colonic mucosal tissue from patients with UC compared to healthy individuals, the pathway and process enrichment analyses were employed to assess the cellular component (CC), biological process (BP), molecular function (MF), and Kegg pathways associated with UC using the Metascape platform (Metascape, http://metascape.org) (31). To identify the MiRGs-driven UC subtypes and their corresponding biological differences, we utilized the MSigDB database (https://www.gsea-msigdb.org/gsea/msigdb/mgs/) and employed the C2: CP: KEGG gene sets and C5: GO gene sets as input files for Gene set Variation Analysis (GSVA) (32). Furthermore, we utilized the single-sample gene set enrichment analysis (ssGSEA) to assess the relative enrichment score of biological pathways across different subtypes. The specific parameters used in this analysis were method = “ssgsea” and kcdf = “Gaussian”.

### 2.8 Protein-protein interaction network construction and module analysis

Utilizing the outcomes of machine learning feature selection, the chosen feature genes were employed to establish a protein-protein interaction (PPI) network using the string database (33), aiming to elucidate the intricate regulatory associations among the corresponding proteins of the feature genes. The visualization of the co-expression network was accomplished utilizing Cytoscape software (V3.10.1) (34). The module for Molecular Complex Detection (MCODE) algorithm was employed to identify hub genes (35). Genes were depicted as nodes, with the size of the shape indicating the MCODE value, while the lines represented the interactions between the genes.

### 2.9 Statistical analysis

Statistical analyses were conducted using R software (version 4.3.2, https://www.r-project.org) and associated R packages. The Kruskal-Wallis test was employed to assess the statistical significance among the three groups, while the Wilcoxon test was utilized to evaluate the statistical significance between the two groups. A significance level of p < 0.05 was deemed statistically significant.

## 3 Results

### 3.1 Identification of DE-MiRGs, functional annotation, and pathway enrichment in UC

**Figure 2:**
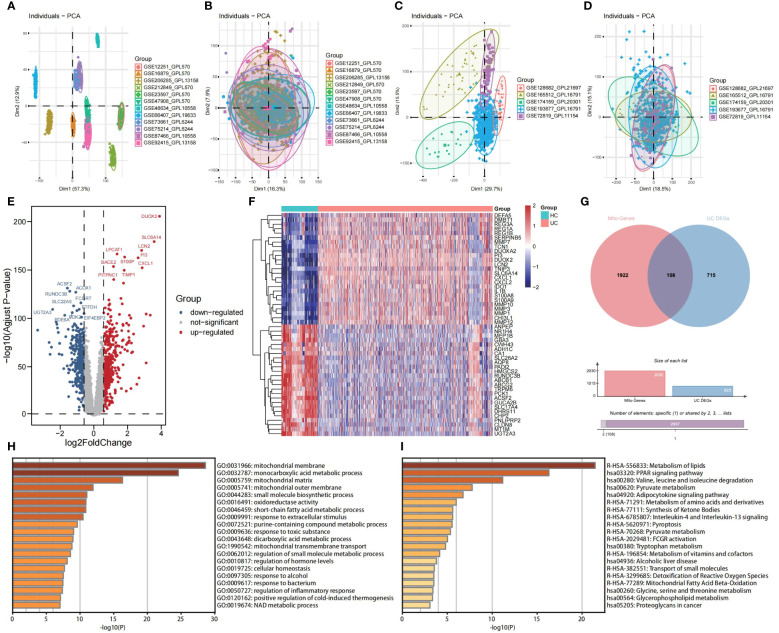
Screening of DE-MiRGs between UC and HC samples, and function enrichment analysis. PCA shows the batch effects of the twelve microarray datasets before **(A)** and after **(B)** the removal of batch effects using the ‘ComBat’ function from the ‘sva’ package in R. PCA shows the batch effects of the five RNA sequencing datasets before **(C)** and after **(D)** the removal of batch effects using the ‘ComBat’ function from the ‘sva’ package in R. Volcano plot **(E)** and heatmap **(F)** show the DEGs in combined GEO microarray datasets of UC and HC in colonic mucosa samples. **(G)** The Venn diagrams to demonstrate the extent of overlapping of DEGs and MiRGs. **(H)** GO enrichment analysis results of DE-MiRGs in BP, CC, and MF were performed with Metascape. **(I)** KEGG and Reactom enrichment analysis results of DE-MiRGs were performed with Metascape.

Principal Component Analysis (PCA) was employed to visually represent the relative distances between datasets, thereby exposing discrepancies between microarray and RNA-seqs datasets utilized in this study (**Figures 2A, C**). Additionally, **Figures 2B, D** demonstrates the effectiveness of the “ComBat” algorithm in eliminating batch effects and data heterogeneity from the integrated microarray datasets and integrated RNA-seqs datasets, respectively. Differential expression analysis of the integrated microarray dataset identified a total of 823 DEGs that exhibited significant differential expression between UC and HC mucosa samples, including 358 down-regulated genes and 465 up-regulated genes in the training cohort (*p* < 0.05, |logFC| > 0.58); results are presented in a volcano plot (**Figure 2E**). The heatmap shown visualizes the expression patterns of the top 20 up-regulated and the top 20 down-regulated DEGs (**Figure 2F**). Subsequently, a combined analysis of 2,030 MiRGs and the 823 DEGs was performed to filter out 108 different expressed MiRGs (DE-MiRGs) in UC mucosa samples, the intersection of which was displayed as a Venn diagram (**Figure 2G**). We used Metascape to carry out GO analysis and KEGG pathway enrichment analysis to explore the potential biological roles of DE-MiRGs in UC. The results of GO enrichment analysis indicated significant involvement in mitochondrial molecular characteristics (mitochondrial membrane and mitochondrial matrix), mitochondrial metabolism of biological process (monocarboxylic acid metabolic process and small molecule biosynthetic process), and oxidoreductase activity (**Figure 2H**). In addition, an investigation of KEGG and the Reactome pathway primarily suggested that these genes were also significantly enriched in immune- and inflammatory-related signaling pathways. These included the peroxisome proliferator-activated receptor signaling pathway and interleukin-4 (IL-4) and interleukin-13 signaling but not mitochondrial energy metabolism, including metabolism of lipids, pyruvate metabolism, and the metabolism of amino acids and derivatives (**Figure 2I**). The terminologies were in alignment with established concepts of UC pathophysiology, that suggests that MiRGs expression patterns participate in the development and progression of UC.

### 3.2 Construction of MiRGs diagnostic models based on machine learning algorithms

**Figure 3:**
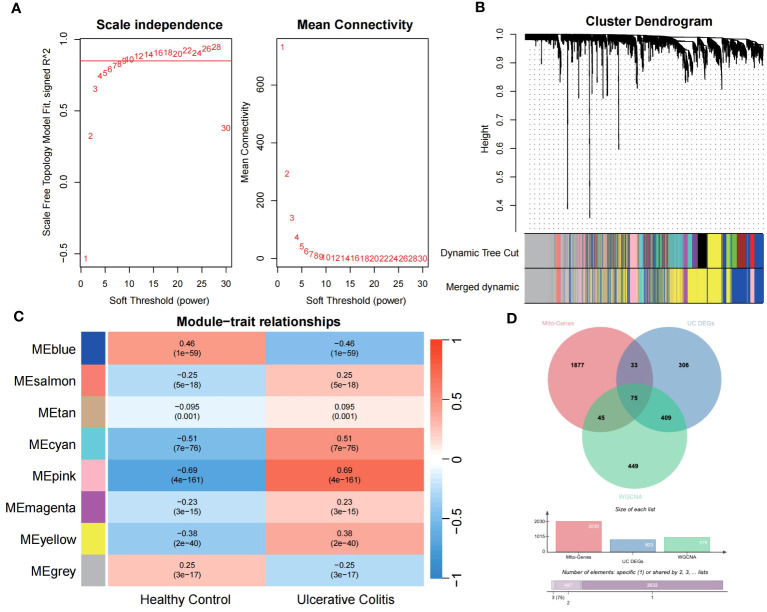
WGCNA reveals modules of co-expressed genes related to UC disease. **(A)** The scale-free fit index for various soft-thresholding powers (β) and the mean connectivity for different soft-thresholding powers determine the final soft threshold of the WGCNA. **(B)** The heatmap of the WGCNA module–trait association. **(C)** The dendrogram displays the clustering of dissimilar genes according to their topological overlap, along with the corresponding merged module colors. **(D)** The Venn diagram shows the common intersection genes of DEGs, module genes identified by WGCNA, and MiRGs.

**Figure 4:**
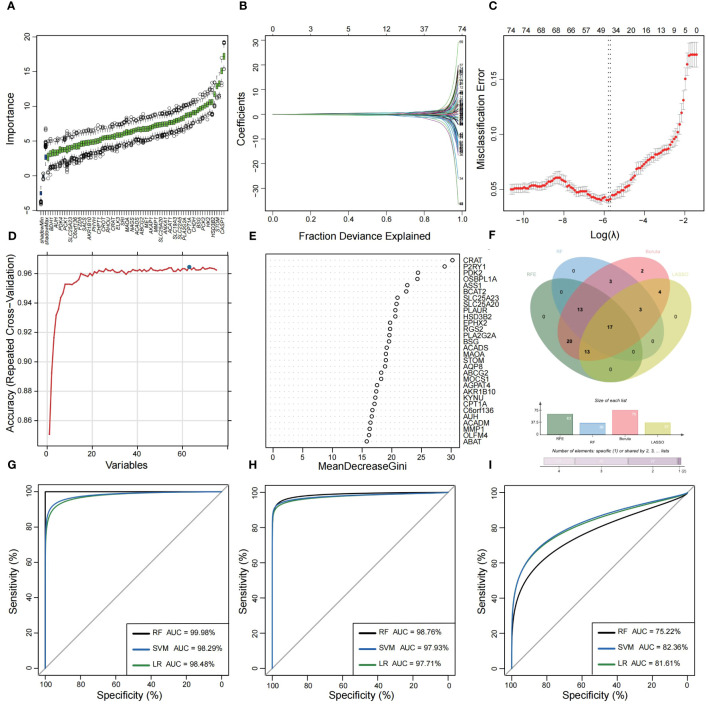
Feature selection and diagnostic model construction via several machine learning algorithms. **(A)** Candidate optimal genes selection based on the Boruta algorithm. The horizontal axis represents the gene, while the vertical axis represents the Z-value of each gene. **(B,C)** The LASSO regression used to select candidate optimal gene for UC. LASSO coefficient profiles of the 75 genes. Optimal parameter (lambda) selection in the LASSO model used tenfold cross-validation via minimum criteria. **(D)** Optimal genes selection using the SVM-RFE algorithm with the highest accuracy and lowest error obtained in the curves. **(E)** Optimal genes identified using the random forest algorithm. Genes importance in random forest assessed by mean decrease in Gini index. **(F)** Venn diagram showed the intersection of Candidate optimal genes identified by the four algorithms. ROC curve of the three machine‐learning diagnosis model based on 17 optimal genes in the training cohort **(G)**, internal test cohort **(H)**, and external validation cohort **(I)**.

Utilizing the WGCNA for module categorization, a total of eight modules were identified (**Figures 3A, B**). We selected the pink module (r = 0.69, *p* = 4e−161), which showed the most positive correlation with UC, and the blue module (r = -0.46, *p* = 1e−59), which showed the most negative correlation with UC as key modules (**Figure 3C**). A total of 978 significant module genes were screened for subsequent analysis, which were overlapped with the 823 DEGs and 2030 MiRGs by the Venn diagram, ultimately yielding 75 candidate genes (**Figure 3D**). Next, four machine learning algorithms [Boruta (**Figure 4A**), LASSO (**Figures 4B, C**), SVM-RFE (**Figure 4D**), and RF (**Figure 4E**)] were used for feature selection in these 75 genes. Finally, we obtained 17 signature genes by taking the intersection of the results of the above four algorithms (**Figure 4F**). Given the current limitations in tools for clinical detection of UC, we constructed diagnostic models using RF, SVM, and LR algorithms utilizing signature genes co-selected by the abovementioned machine learning. The microarray datasets were randomly partitioned into a training cohort and internal test cohort at a ratio of 7:3, while RNA-Seq datasets were used as an external validation cohort. The performance of RF, SVM, and LR models was evaluated in all cohorts using diagnostic ROC curves. In the training cohort, the prediction accuracy of the feature genes screened by the RF algorithm was 99.98%, which was better than those of SVM algorithm (98.29%) and the LR algorithm (98.48%) (**Figure 4G**). Similarly, consistent results are observed in the test cohort that the RF algorithm achieved an AUC value of 98.76%, while SVM and LR models achieved AUC values of 97.93% and 97.71% (**Figure 4H**). Nevertheless, in the validation cohort, AUC values were 75.22% and 82.36%, 81.61%, respectively (**Figure 4I**). Overall, the three machine learning models demonstrate excellent predictive performance, with the SVM model exhibiting the best performance and robustness, which suggests a satisfactory diagnostic effectiveness of 17 MiRGs for a clinically precise diagnosis of UC.

### 3.3 Immune-infiltrating landscape of UC

**Figure 5:**
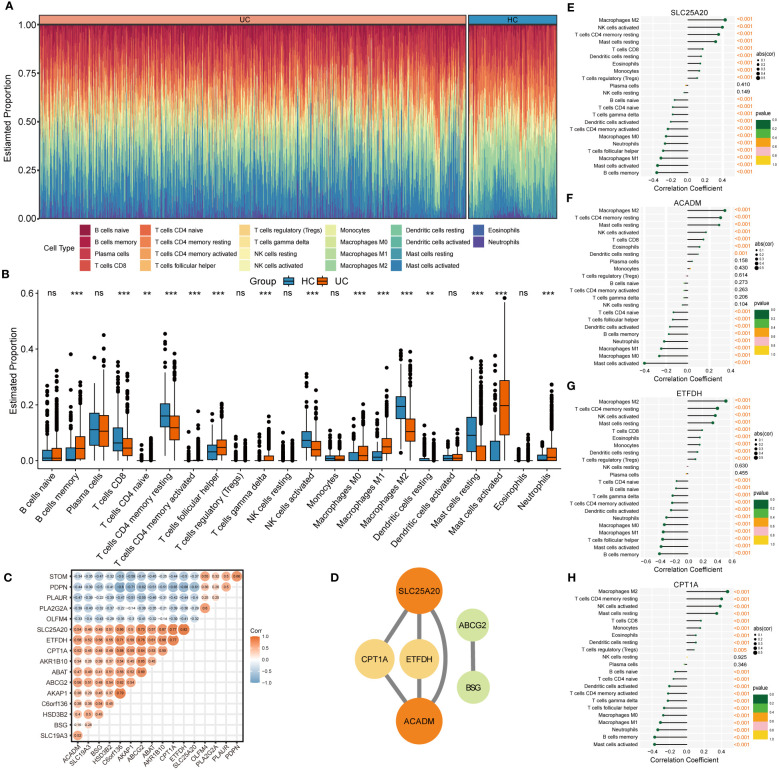
Immune infiltration analysis of UC. **(A)** Histogram showing the distribution of 22 immune cells infiltration between the UC samples and HC samples. **(B)** The Boxplots represented proportion of the 22 typical immune cells infiltrating in the colonic mucosa immune microenvironment of UC samples and HC samples based on the CIBERSORT algorithm. ns, not significant; **p <0.01; ***p<0.001. **(C)** Heatmap showing statistically significant correlations between 17 optimal genes identified by the four machine‐learning algorithms based on Spearman’s correlation. **(D)** Further identification of hub genes from the PPI network by using the MCODE algorithm. **(E-H)** The lollipop plots showing the correlations between 4 hub genes and 22 typical immune cells.

The CIBERSORT algorithm was employed to characterize the abundance of 22 immune cell infiltration in the colonic mucosa of UC and HC groups. **Figure 5A** shows the distribution of immune cell types in each sample for both groups, while **Figure 5B** illustrates differences in the abundance of infiltrated immune cells between the two groups. Compared with HC groups, higher levels of memory B cells, follicular helper T cells, γ & δ T cells, M0 and M1 macrophages, activated dendritic cells, and activated mast cells and neutrophils were found to be infiltrating the colonic mucosa of patients with UC. This indicated a significant difference in the immune microenvironment between UC and HC groups. Spearman’s correlation analysis was used to investigate the relationship among the 17 signature genes. We found that SLC25A20, ACADM, ETFDH, SLC19A3, CPT1A, BSG, and other genes exhibited predominantly positive correlations, while STOM, PDPN, PLAUR, PLA2G2A, and OLFM4 showed mainly negative correlations (**Figure 5C**). Subsequently, we constructed a PPI network of these signature genes using a STRING database to reflect their interaction. The result was visualized by Cytoscape, as shown in **Figure 5D**, in which four highly related hub genes were identified, including SLC25A20, ACADM, CPT1A and ETFDH. To clarify the potential association between hub genes and UC, we assessed the correlation between the expression levels of four hub genes and 22 immune-infiltrating cells in the colonic mucosa of patients with UC. The result demonstrated that all four hub genes showed a variety of correlation with immune infiltration. Such hub genes exhibited a close negative link with most immune cells, such as activated mast cells, memory B cells, M0 and M1 macrophages, and activated DCs. The hub genes were positively associated with M2 macrophages, activated NK cells, resting CD4 memory T cells, resting mast cells, and CD8 T cells (**Figures 5E–H**). This finding is consistent with our previous studies, and may facilitate understanding the immunological features and molecular mechanisms of UC.

### 3.4 Identification of three molecular subtypes of UC with distinct cellular and molecular features driven by MiRGs

**Figure 6:**
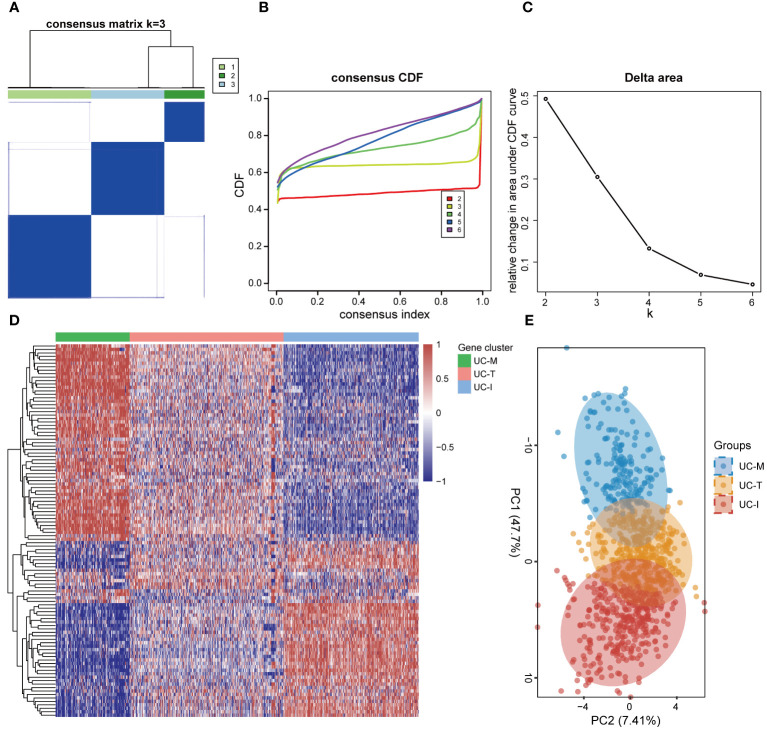
Unsupervised consensus clustering identified the subtype of UC driven by MiRGs. **(A)** Consensus matrix heatmap defining three clusters. The bars between the dendrogram and the heatmap represent the molecular clusters. A stable and robust clustering of the samples is evident from the boundary of the consensus matrix. **(B)** The Cumulative distribution function (CDF) curve of different K-values. When k = 3, the CDF curve with the slowest downward trend represents the most stable clustering. **(C)** The Delta area plot of different K-values, indicating the relative change in area under the CDF curve between K and K-1. **(D)** Gene expression heatmaps of 941 UC samples based on consensus cluster assignment. The colorful scale of heatmap reflects the relative expression levels where blue represents low expression and red represents high expression. **(E)** Principal components analysis (PCA) for the DE-MiRGs expression profiles showing the stability and reliability of the classification.

In order to establish a more comprehensive definition of mitochondria-related gene expression-driven subgroups in UC, we performed an unsupervised cluster analysis on 941 UC colonic mucosal samples based on 108 DE-MiRGs. As a result, k = 3 was identified as the optimal number of clusters to ensure the robustness of clustering results, based on the CDF values and delta area (**Figures 6A–C**). Consequently, we finally obtained three MiRGs driven subtypes of UC, designated as UC-M (n = 192), UC-T (n = 399), and UC-I (n = 350) subtypes. The heatmap showed obvious heterogeneity in the gene expression profiles of these 108 DE-MiRGs among the three subtypes (**Figure 6D**). The PCA results further confirmed a distinct separation between the three UC subgroups (**Figure 6E**).

**Figure 7:**
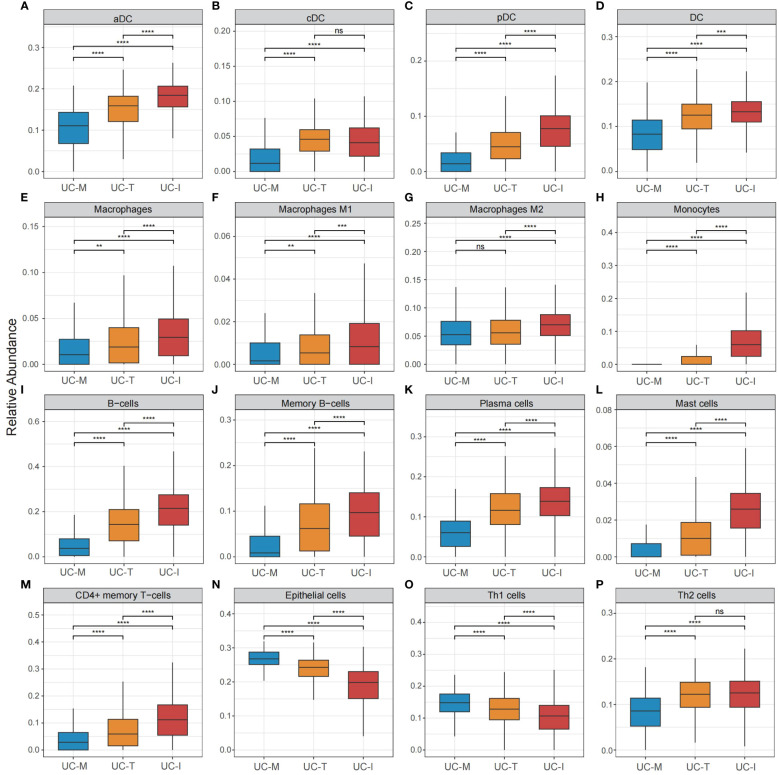
Cell subpopulation-driven characterization of UC subtypes. **(A–P)** Box plots revealed cell subpopulation enrichment scores across the UC colonic mucosa subgroups. Differences across the three subgroups were analyzed using the Wilcoxon test; ns, not significant; **p <0.01; ***p <0.001; **** p<0.0001.

**Figure 8:**
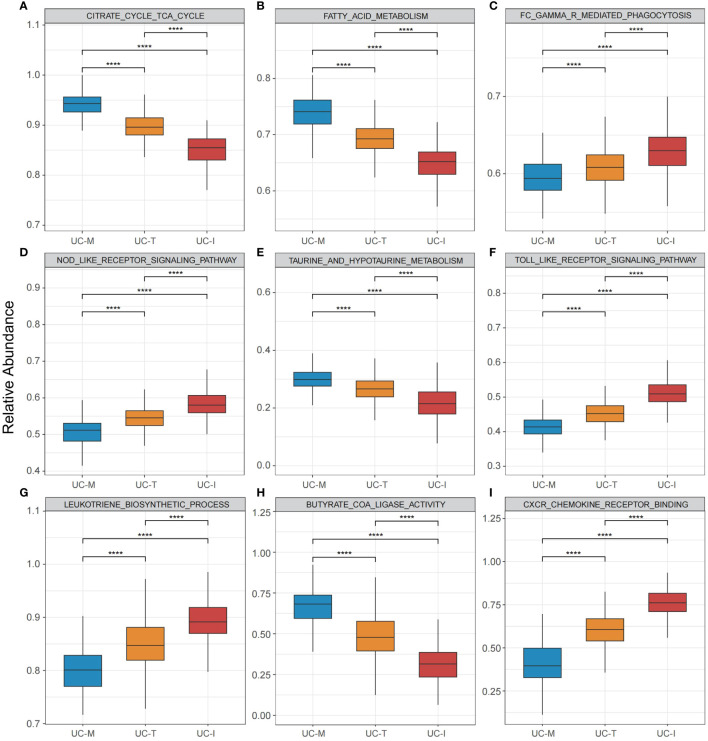
Pathway-driven characterization of UC subtypes. **(A–I)** Box plots revealed pathway activation scores across the UC colonic mucosa subgroups. Differences across the three subgroups were analyzed using the Wilcoxon test; **** p < 0.0001.

To explore the possible molecular attributes and physiological roles of three subtypes in the colonic mucosa of patients with UC, we characterized different MiRGs related to the immune state across 64 cell signatures and immune-related pathways. The results of differential abundance of immune cell infiltration revealed that the UC-I subtype was highly infiltrated by most immune cells, especially antigen-presenting cells (including DCs, monocytes, macrophages, and B cells), while the UC-M subtype was distinctly enriched in epithelial cells (**Figure 7**). GSVA analysis was performed to assess differences in functions and pathways enriched in mitochondria gene expression-driven subtypes. **Figure 8** shows that the UC-M subtype had a relatively high enrichment score in metabolism-related pathways. For instance, these included the citrate cycle (tricarboxylic acid [TCA] cycle), butyrate CoA ligase activity, fatty acid metabolism, and taurine and hypotaurine metabolism. In comparison, immune- and inflammation-related pathways, such as CXCR chemokine receptor binding, the NOD-like receptor signaling pathway, Toll-like receptor signaling pathway, and FCγR-mediated phagocytosis, were significantly enriched in UC-I subtype. Most immune cells and immune-related pathways showed modest activation in the UC-T subtype. These results suggest significant differences in immune infiltration, biological function, and pathway activity between different UC subtypes. Thus, utilizing these molecular features, the UC-M subtype was defined as a metabolic subtype enriched in epithelial cells, the UC-T subtype was described as a transitional subtype, and the UC-I subtype was defined as an immune-inflamed subtype enriched in antigen-presenting cells. In addition, DCs showed particular infiltration in this study, so their role in UC deserves further investigation.

### 3.5 Validation of classification by external cohort

The robustness of classification results was confirmed by integrating five publicly available UC mucosal biopsy RNA-seq datasets. Patients were categorized into three subtypes using the gene expression profiles of 108 DE-MiRGs (designated as UC-M (n = 283), UC-T (n = 291), and UC-I (n = 180) subtypes (**Supplementary Figure 1**). Our findings were consistent with the enrichment scores of UC-related cell subpopulations and pathways (**Supplementary Figure 2**,**3**). The UC-M subtype was characterized by epithelial proliferation and mitochondrial metabolism, UC-T was described as showing modest immune activation, and UC-I was identified as an immune-inflamed type.

### 3.6 The efficacy of biological agents was significantly different between the three UC subtypes

**Figure 9:**
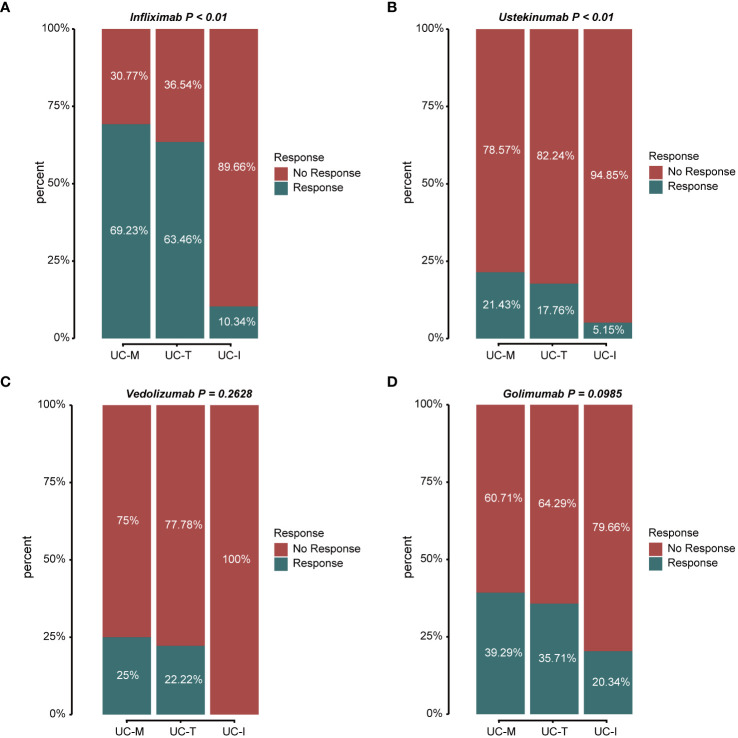
Stacked bar graphs showing the response status of different subtypes to multiple biological agents. Response: responded to the biologics; non-response: did not respond to the biologics. **(A)** Response/non-response to IFX: 69.23%/30.77% in subtype UC-M, 63.46%/36.54% in subtype UC-T, and 10.34%/89.66% in subtype UC-I. **(B)** Response/non-response to UST: 21.43%/78.57% in subtype UC-M, 17.76%/82.24% in subtype UC-T, and 5.15%/94.85% in subtype UC-I. **(C)** Response/non-response to VDZ: 25.00%/75.00% in subtype UC-M, 22.22%/77.78% in subtype UC-T, and 0%/100% in subtype UC-I. **(D)** Response/non-response to GLM: 39.29%/60.71% in subtype UC-M, 35.71%/64.29% in subtype UC-T, and 20.34%/79.66% in subtype UC-I.

According to the latest IBD Treatment Guidelines 2023, biological agents such as the tumor necrosis factor (TNF)-α inhibitors, infliximab (IFX) and Golimumab (GLM), anti-α4β7 integrin antibodies, vedolizumab (VDZ), and the IL-12/IL-23 inhibitor, ustekinumab (UST), are approved for the treatment of UC and have been proposed as first-line treatment options for moderate to severe UC. Thus, we evaluated the response to treatment in different subtypes using the above four biologics (**Figures 9A–D**). The proportion of favorable responses observed for the UC-M and UC-T subtypes consistently exceeded that of the UC-I subtype. All biological agents showed the highest response rates in the UC-M subtype. It is worth mentioning that the UC-M [69.23% and 39.29%] and UC-T [63.46% and 35.71%] subtypes exhibited favorable responses for IFX and GLM, while the UC-I subtype was completely unresponsive to VDZ. Moreover, UST elicited relatively inadequate responses in all three subtypes. However, due to insufficient sample size, these differences may not have reached statistical significance. In conclusion, the efficacy of biological agents in UC patients may be influenced by the unique pathological characteristics of individual colonic mucosal tissues and their molecular activity. Our study indicates that the diverse molecular subtypes of colonic mucosa in ulcerative colitis could impact the response to drug therapy, highlighting the importance of considering these factors in the clinical application of these medications.

## 4 Discussion

Emerging evidence indicates that mitochondrial dysfunction plays a role in the progression of UC by affecting the integrity of the intestinal epithelial barrier and mucosal immune tolerance. This study establishes a connection between mitochondrial-related genes and UC, investigates potential pathogenic mechanisms, and presents novel avenues for the diagnosis, categorization, and management of the disease.

By conducting a thorough bioinformatics analysis of the largest cohort of patients with UC to date, we identified 17 signature genes associated with mitochondria that play a significant role in the progression of the UC. We developed genetic diagnostic models for UC using multiple machine learning algorithms that demonstrated strong predictive capabilities and clinical utility. Furthermore, a systematic bioinformatic analysis was performed on the screened genes to investigate the intrinsic relationship between gene-gene and gene-disease. Our study identified a significant correlation, either positive or negative, among the signature genes, indicating potential synergistic or antagonistic interactions that may contribute to the development and advancement of ulcerative colitis. Four hub genes (SLC25A20, ACADM, CPT1A and ETFDH) were subsequently identified by PPI network analysis.

As of now, there has been no comprehensive investigation into the role of UC resulting from the activity of these four genes. CPT1A, identified as a promising target for clinical therapy, catalyzes the transfer of a long-chain acyl group from an acyl-CoA ester to carnitine, allowing fatty acids to enter the mitochondrial matrix for oxidation (36). The DSS-induced mice model of UC has been reported that downregulation of CPT1A can protect UC partially by inhibiting PPARα signaling, which suggest that the development of small molecule drugs targeting CPT1A may provide prospective therapeutic options for the UC clinical therapy (37). Additionally, a Mendelian randomization analysis of the specific MiRGs in IBD (38) indicated that genetically predicted levels of ACADM methylation, expression, and the corresponding protein were highly correlated with UC. Nevertheless, there is limited evidence from observational epidemiological and experimental studies regarding the association between ACADM and UC. Thus, ACADM is prioritized as a potential drug target for UC, and needs to be validated in future trials. For inflammatory UC, the study by Jan Söderman et al. showed that SLC25A20 has been documented downregulation in inflamed UC mucosa but upregulation was detected in non-inflamed UC mucosa (39). Therefore, we suggest that targeting SLC25A20 in pharmacological application of our UC-I subtype has great potential. A significant role for feasible therapy against ETFDH in UC has not been reported. In the future, we will try animal experiments or other measures to explore the role of ETFDH in UC and the mechanisms of these four hub genes in UC treatment. Our research will also focus on elucidating the underlying drug-gene interactions in order to identify potential therapeutic agents. Currently, anti-TNF-α drugs and other biological agents have been used in UC for many years. It is urgent and essential to develop specific drugs targeting MiRGs for refractory patients who do not show an objective treatment response.

According to reports from the last few years, mitochondrial dysfunction may underlie intestinal mucosal injury (40). Mitochondrial metabolism is key to the regulation of the function of the intestinal epithelial barrier function. For instance, due to defective mitochondrial acetoacetyl CoA thiolase activity in UC, disrupted β-oxidation of butyrate, the preferred energy source of colonic epithelial cells (41), as first shown by Roediger (42), has been implicated in the pathogenesis of UC. However, mitochondrial dysfunction induces inflammatory responses in innate immune cells by promoting the production of pro-inflammatory cytokines (TNF-α, IL-1β, and interferon [IFN]-γ) through excessive derived reactive oxygen species and an active endogenous damage-associated molecular pattern (43–46). In addition, mitochondrial metabolism is also intimately linked to immune inflammation. Studies have shown that the mitochondrial metabolic state of immune cells can influence their phenotype, pro-inflammatory or anti-inflammatory polarization states, and the efficacy of immune responses (17). In the case of macrophages, M1 macrophage polarization depends on anaerobic glycolysis, whereas M2 macrophage polarization relies on fatty acid metabolism and OXPHOS (47). These are consistent with our subtype classifications driven by mitochondrial gene expression. Specifically, the UC-M subtype has a transcriptomic signature in epithelial cell proliferative-related pathways, while the IC-I subtype exhibits high enrichment in immune cells and proinflammatory activation-related pathways. Expectedly, the UC-T subtype is considered a transitional subtype.

The crucial role of the immune response in the UC pathogenesis has been the focus of much attention. Our systematic evaluation of immune infiltration revealed that the expression levels of almost all immune cells in UC were significantly higher than those in the HC cohort, which is consistent with the results of many previous studies. Magnusson et al. (48) found that increased numbers of DCs and macrophages, activated upon Toll-like receptors, are characterize the inflamed intestinal lamina propria in UC. This is consistent with our observation of immune cell infiltration in an immune-inflamed subtype. Notably, the metabolism of DC subsets is intrinsically linked to their ability to control Th cell polarization (49). Gut cDC1 produces more IL-12 to drive Th1 polarization than any other DC subsets especially cDC2, which is responsible for Th2 differentiation in a variety of type 2 immune responses (50). Paradoxically, high Th1 expression was observed in the metabolic subtype in our study. This could possibly because the TCA cycle supports Th1 cell proliferation and function through distinct mechanisms. An example is promoting the production of IFN-γ by elevating cytosolic acetyl-CoA pools through mitochondrial citrate export, which cooperated with TNF-α to induce apoptosis of intestinal epithelial cells and damage to the intestinal mucosal barrier (51, 52). Moreover, IL-12 expression of DCs has been demonstrated to be critically dependent on glycolysis (49). Such prior studies all strongly support our findings. We further speculate that DCs with different Th cell-polarizing properties have distinct mitochondrial metabolic profiles. Additionally, gut DCs have been reported to induce more IL-4 (Th2 cytokine) production but less IFN-γ (Th1 cytokine) and less IL-22 production by T cells. This leads to an enhanced capacity to imprint gut-specific homing properties on effector T cells, skewing gut-specific T cell responses towards a Th2 profile (53, 54). This supports the evidence of UC being a Th2-dominant disease.

In light of our analysis, the metabolic subtype exhibited a higher abundance of epithelial cells and exhibited favorable responses to diverse pharmacological interventions. Whereas the immune-inflamed subtype, characterized by antigen-presenting cell infiltration, presented divergent outcomes and demonstrated inadequate responsiveness to multiple medications. The observed variations in disease activity and drug efficacy across distinct subtypes hold promising clinical implications for the management of UC in patients. The ability of IL-22 to promote stem cell–mediated intestinal epithelial regeneration in mice and humans has been repeatedly demonstrated by Lindemans et al. (55) using different experimental approaches. Others have shown the transfer of IL-22 producing immune cells can enhance recovery from dextran sodium sulfate-induced (56, 57). In addition, further studies revealed that increased IL-22 production after IFX therapy responded well to epithelial cell repair (58). Besides its anti-inflammatory properties, IFX can also enhance the glycerophosphatidylcholine level by regulating lipid metabolism to protect the integrity of intestinal mucosa (59, 60). These findings highlight the superior outcomes of anti-TNFα agents in the UC metabolic subtype.

The immune-inflamed subtype exhibited a less-than-ideal response to various drugs, with a secondary loss of response. The reason for this mechanistic escape remains unclear but is thought to be primarily immunogenicity or an inflammatory burden, resulting in increased drug clearance (61–63). It was deduced that patients exhibiting immune-active characteristics and high levels of immune inflammation are likely to have refractory UC and may warrant therapeutic drug monitoring. For such refractory patients, combining targeted therapies (CTT) may act synergistically to enhance the response to therapy, cover extraintestinal manifestations of disease, or reduce the risk of treatment failure (64). IFX has more potent immunogenicity than other anti-TNF-α agents, because IFX is a chimeric antibody of which 25% has a murine structure (63). In the UC-SUCCESS randomized double-blind trial (65), patients receiving combination therapy with IFX and azathioprine (39.7%) had a significantly higher remission rate at week 16, compared with IFX alone (22.1%). Patients also showed reduced antibody formation against IFX, but had an increased risk of infection and tumor induction. Not enough evidence exists to support the combination of immunosuppressants with other anti-TNF-α agents such as GLM. The gut-selective properties of VDZ would theoretically support its selection as one of the options for combination regimens to reduce the risk of adverse events (63). Nevertheless, the TREAT registry dose not indicated any benefit in a combination of immunosuppressants with VDZ or UST. Hence, combining small molecule agents targeting specific inflammatory pathways may be one of the most promising emerging therapeutic options for patients with refractory UC who lack effective alternatives. Tofacitinib, a pan-active small molecule pan-active JAK inhibitor, has been demonstrated to inhibit the inflammatory process in UC, mainly by mediating the JAK/STAT inflammatory signaling and pro-inflammatory cytokine signaling, such as by IL-6 and IFN-γ (66, 67). It is also an attractive choice as an oral agent without concerns about being immunogenic. The efficacy and safety of tofacitinib have been confirmed in clinical trials and real-world practice in the maintenance treatment of refractory UC patients (68–70). Recent retrospective studies by Gilmore et al. (71) and Hilley et al. (72) reported that all patients with refractory UC who showed a partial response to a single biologic agent remained on biologics/tofacitinib combination therapy. Of this, 71% vs. 80%, respectively, achieved clinical remission, and 43% vs 40%, respectively, achieved endoscopic remission; clinical response rates were both higher than with monotherapy. In addition, apremilast, a new oral small-molecule PDE4 inhibitor, can cause an increase in intracellular cAMP levels to reduce those of pro-inflammatory mediators (e.g., TNF-α and IL-23, activating DCs) and increase levels of anti-inflammatory mediators (e.g., IL-10, inhibiting DCs) (73). A recent 12-week phase II clinical trial (No. NCT02289417) conducted by Danese et al. (74) revealed improvements in multiple efficacy indicators in patients with active UC treated with apremilast. Together with our above analysis, we suggest that apremilast functions as a safe and well-tolerated anti-inflammatory agent, targeting DCs specifically, for patients with UC. The therapeutic response in combination with biological agents still needs to be validated in future trials. That is, biologics/small molecule agent combination therapy, to the extent that it reduces both immunogenicity and a high inflammatory burden, has the potential to minimize steroid exposure, induce remission, and avoid colectomy by facilitating a safe transition to maintenance therapy. However, relevant research is still limited. Rigorous randomized trials are needed to obtain efficacy and safety data as well as to assess and carefully discuss risks and benefits for short durations to minimize the risk of adverse events. Hence, further investigation is warranted to assess the efficacy of combining biologics with small molecule inhibitors in the treatment of refractory ulcerative colitis, potentially yielding valuable insights for managing patients with immune-inflammatory subtypes of the disease.

Nonetheless, several limitations in this study bear mentioning. First of all, more metadata would be ideal, but an unavoidable selection bias may exist. Second the complete annotation of clinical information for each UC sample is lacking. Furthermore, we found that the robustness of the diagnostic model in the validation cohort was worse than that in the training and testing cohorts (82.36%% vs 97.93%/98.29%%). The main reason for this discrepancy may be the different data sources that the clinical samples in the internal training and testing cohorts were from microarray datasets, while RNA-seq datasets was applied in the external validation cohort. However, this bias does not affect our results. Considering the above results, our study was conducted using only clinical samples, without *in vivo* or *in vitro* validation. Therefore, further studies are required to provide convincing evidence for our results, which will be the focus of our future research.

## 5 Conclusions

In conclusion, this is the first and largest study published to date on the diagnosis and classification of UC from the perspective of mitochondrial dysfunction. We employed bioinformatics methods to identify signature genes of high value, and developed a predictive model with precise diagnostic capabilities. Furthermore, we categorized ulcerative colitis into three distinct groups using MiRGs. Our findings can be used to better elucidate the heterogeneity and treatment response of patients with UC in order to gain an insight into molecular mechanisms and to design stratified treatment protocols for such patients.

## Data availability statement

The datasets presented in this study can be found in online repositories. The names of the repository/repositories and accession number(s) can be found in the article/**Supplementary Material**.

## Author contributions

Y-fZ: Data curation, Methodology, Supervision, Writing – original draft, Writing – review & editing. M-yF: Writing – original draft, Writing – review & editing. Q-rB: Data curation, Writing – review & editing. RZ: Data curation, Writing – review & editing. SS: Data curation, Writing – review & editing. LW: Data curation, Writing – review & editing. J-hL: Visualization, Writing – review & editing. J-wL: Visualization, Writing – review & editing. QW: Data curation, Writing – review & editing. YL: Data curation, Writing – review & editing. XC: Writing – review & editing.
