# Supplementary material for: Precision therapy for ulcerative colitis: insights from mitochondrial dysfunction interacting with the immune microenvironment
Source: Front Immunol. 2024 Jul 4;15:1396221. doi: 10.3389/fimmu.2024.1396221 (PMC11254623; doi:10.3389/fimmu.2024.1396221)
Supplement: Supplementary file 1 [file DataSheet_1.zip › Supplementary_Material.docx]

Supplementary Material

# Supplementary Figures and Tables

## Supplementary Figures


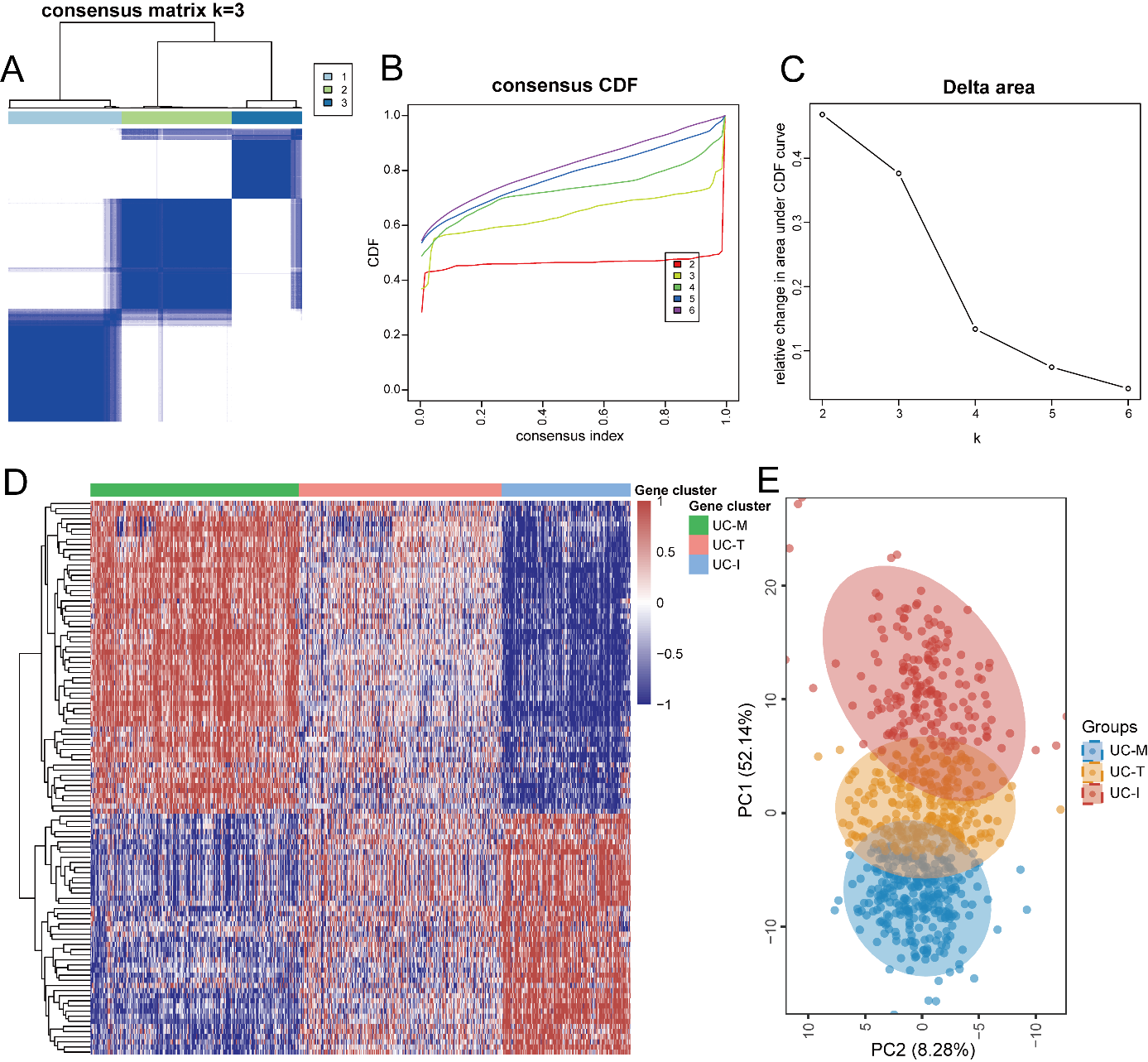


**Supplementary Figure 1.** External validation of the subtype of UC driven by MiRGs.


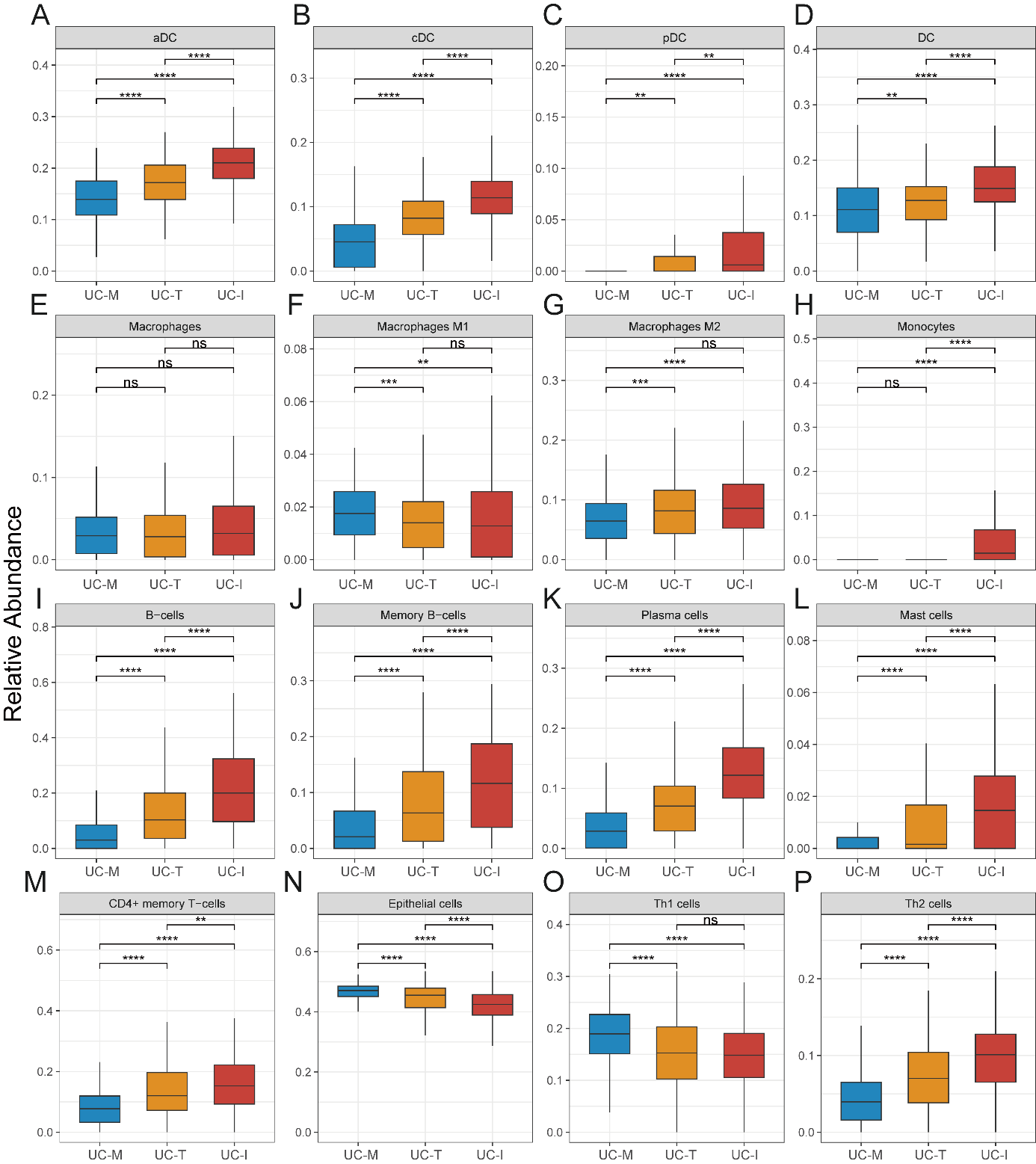
 **Supplementary Figure 2.** Cell subpopulation-driven characterization of UC subtypes on the validation cohort.


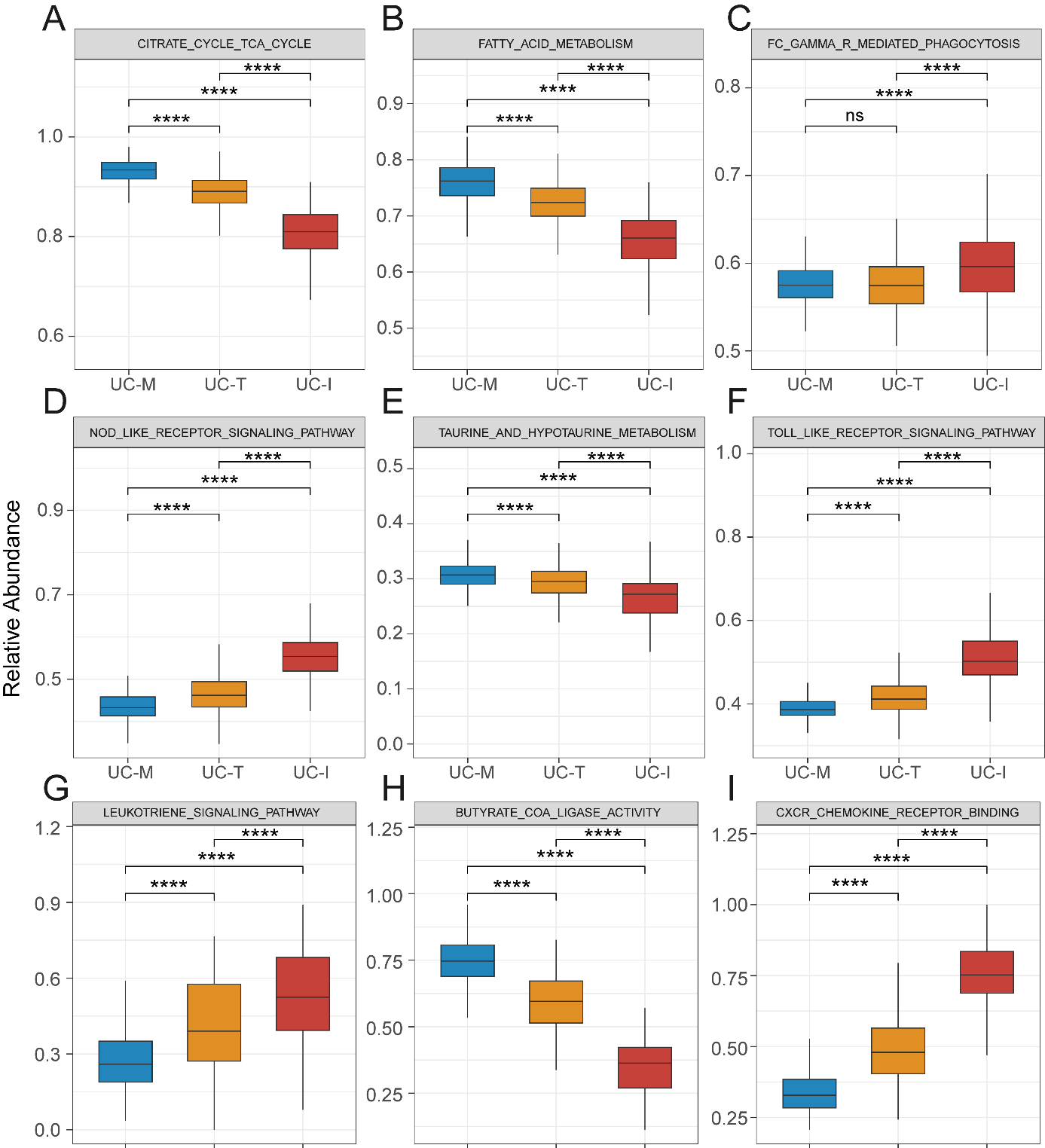


**Supplementary Figure 3.** Pathway-driven characterization of UC subtypes on the validation cohort.

## Supplementary Tables

**Supplementary Table 1.** Summary information of patients with UC.

**Supplementary Table 2.** Mitochondrial genes (n = 2030) and mitochondria-related GSEA gene sets (n = 163).

**Supplementary Table 3.** All DEGs in UC and HC groups.

**Supplementary Table 4.** All Genes identified by WGCNA.

**Supplementary Table 5.** Intersection results of four machine learning algorithms, Random Forest, SVM-RFE, LASSO and Boruta.
